# Ulnar collateral ligament injuries of the first metacarpophalangeal joint: prevalence of associated injuries on radiographs and MRI

**DOI:** 10.1007/s00256-020-03575-w

**Published:** 2020-08-20

**Authors:** Sebastian Manneck, Filippo Del Grande, Anna Hirschmann

**Affiliations:** 1 Clinic of Radiology and Nuclear Medicine, University of Basel Hospital, Petersgraben 4, 4031 Basel, Switzerland; 2 Department of Radiology, Ospedale Regionale di Lugano, 6900 Lugano, Switzerland

**Keywords:** Ulnar collateral ligament, Volar plate, Thumb, Metacarpophalangeal joint, MRI, Hand injuries, Radiographs

## Abstract

**Purpose:**

To evaluate the prevalence of associated findings at the first metacarpophalangeal joint on radiographs and MRI following acute ulnar collateral ligament (UCL) injuries.

**Materials and methods:**

This retrospective study included 25 patients with an injury of the UCL at MRI. Presence of associated injuries to the volar ligaments (checkrein and phalangoglenoid ligaments and volar plate) was assessed on radiographs and MRI independently by two musculoskeletal radiologists. Wilcoxon signed-rank test was used to compare frequencies of injuries between both modalities (*p* < 0.05). Interreader variability was calculated.

**Results:**

Complete tears of the UCL (48%/60%, reader 1/2) were more common than partial tears (24%/16%) on MRI. Dislocation of the UCL ≥ 3 mm was detected in 40%/56% on MRI. UCL avulsion fractures were more frequently seen on MRI (28%) compared with radiographs (12%) for reader 1. Associated avulsion injuries of the phalangoglenoid ligament were evident in 12%/8% on radiographs and in 80%/76% on MRI. Almost all patients (100%/79%) with a dislocated UCL tear showed a concomitant volar ligament injury; and even two-thirds (66%/72%) of the non-displaced UCL tears had an injury to the volar ligaments. Interreader agreement was moderate to excellent (*κ* = 0.60–1.0).

**Conclusion:**

UCL tears are often associated with volar ligament injuries, even in lesser degrees of an UCL injury.

## Introduction

The ulnar collateral ligament (UCL) is the primary stabilizer of the first metacarpophalangeal (MCP) joint during valgus stress. Injuries of the UCL typically occur as a result of hyperabduction and may be accompanied by hyperextension of the thumb. UCL tears most commonly occur at the distal insertion and the stump may be displaced. Surgical therapy is recommended if the displacement is more than 3 mm [1] and required if the displacement is proximal to the adductor aponeurosis, also known as Stener lesion [2].

**Fig. 1:**
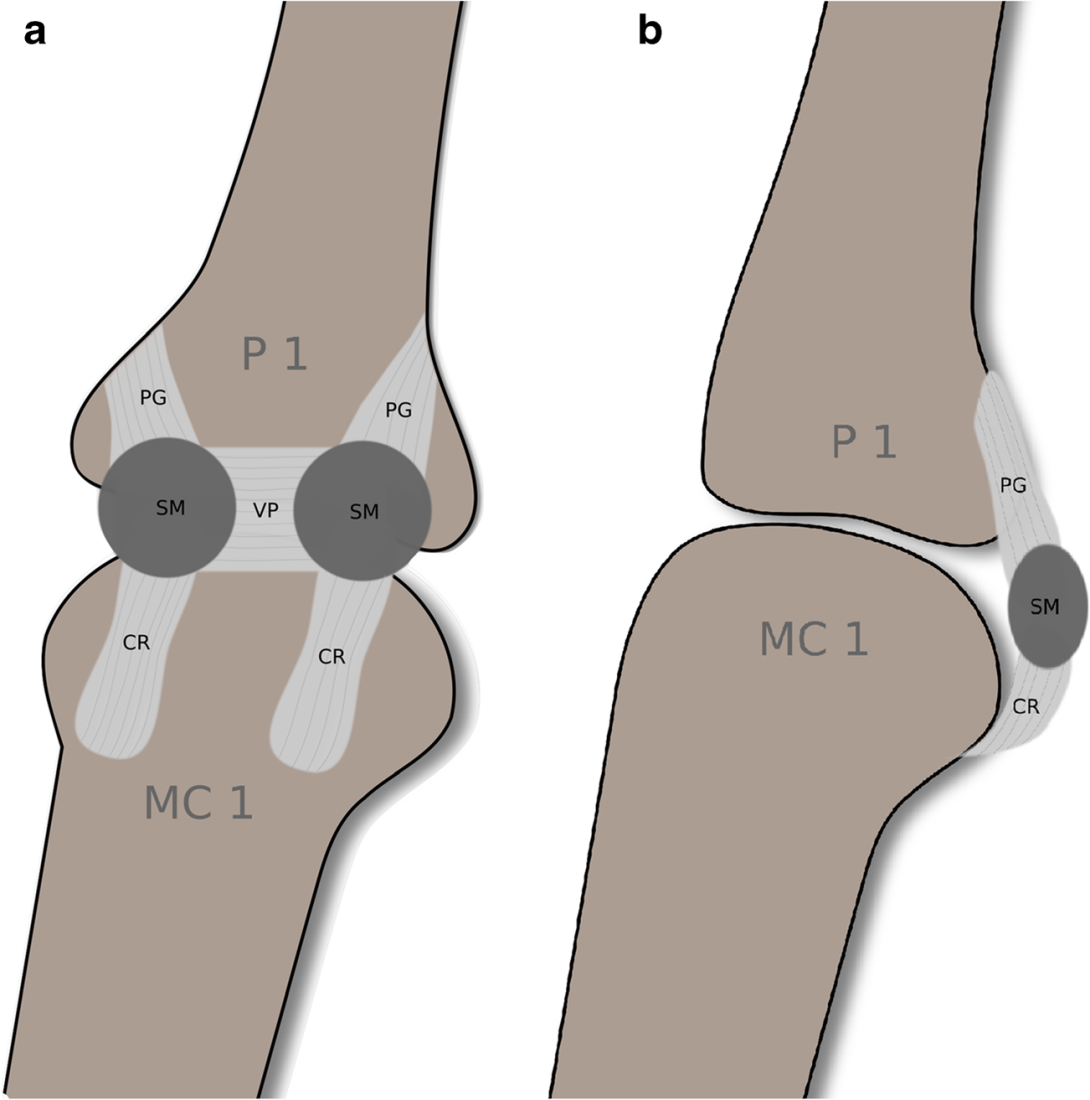
The first metacarpophalangeal joint and volar ligaments in coronal (**a**) and sagittal (**b**) planes. CR checkrein ligaments, SM sesamoid bones, VP volar plate (also known as intersesamoid fibers), PG ulnar and radial phalangoglenoidal ligaments

A misdiagnosed or improperly treated injury may result in joint instability, leading to significant disability and pain and may provoke early osteoarthritis of the first MCP joint [3]. The volar ligaments of the first MCP joint reinforce the volar capsule deep to the flexor tendon [4]. Three components comprise the volar ligaments: the ulnar and radial checkrein ligaments, the ulnar and radial phalangoglenoid ligaments, and transverse intersesamoid fibers; the latter is also known as the volar plate [5–7]. The checkrein ligaments insert proximally at the head of the metacarpal bone and embed the ulnar and radial sesamoid bones. The phalangoglenoid ligaments originate at the ulnar and radial sesamoids and traverse in an oblique fashion distally inserting at the outer base of the proximal phalanx. The volar plate connects both sesamoid bones and has a broad distal insertion at the center of the base of the proximal phalanx (Fig. 1) [6, 7].

The term “volar or palmar plate” is inconsistently used for either the intersesamoid fibers [5–8] or the phalangoglenoid ligaments [3] and the term “volar plate injury” is used without specifying the ligaments in detail [3, 8–12]. Therefore, and in line with Stener [2], we summarized the three ligaments (checkrein ligaments, phalangoglenoid ligaments, and volar plate) to the term “volar ligaments” to eliminate misunderstanding. The volar ligaments together with the sesamoids are an important stabilizer in extension and limit hyperextension [1]. Post-traumatic hyperextension with an unrecognized or improperly treated tear of the volar ligaments may lead to instability [13].

Due to the commonly combined trauma mechanisms of hyperabduction and hyperextension, associated injuries to the volar ligamentous complex and the dorsoulnar capsule may occur [8, 14], indicating a more severe joint instability with the need of an extensive surgical approach. However, until today, no validated data have been available displaying the prevalence of associated soft tissue injuries about the first MCP joint in patients suffering from UCL tears.

Radiography usually is the first-line imaging modality following an acute trauma of the first MCP joint to investigate avulsion fractures and joint incongruities, although radiographs may be negative. Inconclusive physical examinations may warrant ultrasound and magnetic resonance imaging (MRI) with the potential benefit to display the full extent of ligament injuries. Both modalities, ultrasound and MRI, are equally applicable in thumb injuries and depend mostly on institutional preferences and availabilities [15, 16]. MRI has the potential benefit to objectively display all structures about the first MCP joint.

The purpose of this study was to evaluate the prevalence of associated findings at the first MCP joint on radiographs and MRI following acute UCL injuries.

## Materials and methods

This retrospective single-center study was approved by the institutional review board.

### Patient selection

**Fig. 2:**
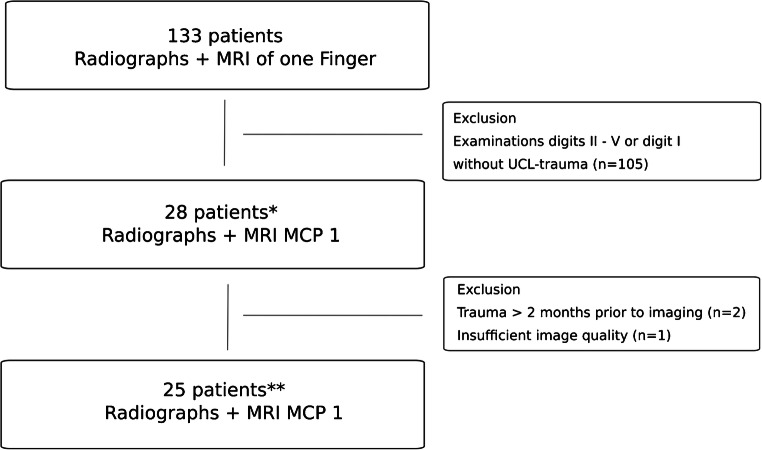
Flowchart shows selection of patients. Examinations were included between January 2014 and November 2018. MRI magnetic resonance imaging, MCP metacarpophalangeal, UCL ulnar collateral ligament. *Patients with trauma to the ulnar collateral ligament according to the original radiology report

All patients, at least 18 years old, with radiographs and MR images of one finger acquired between January 2014 and November 2018, were selected through a search of our radiology information system database. The resulting 133 patients were reviewed manually for radiographs and MR examinations of the thumb and for injury of the UCL by reading the radiology reports. Figure 2 demonstrates the selection of patients. The medical records of the remaining 28 patients were investigated for a trauma setting and the time interval between imaging by one musculoskeletal fellow radiologist.

Patients with evidence of sprain or rupture of the UCL on MR images, a time interval of up to 6 weeks between radiographs and MRI, and imaging within 2 months of trauma were included in this study. Three patients were excluded due to the following reasons: two for having a time interval of more than 2 months between imaging and trauma, and one for insufficient image quality on MRI. Twenty-five patients met the inclusion criteria.

### Imaging technique

Radiographs included a lateral and anterior-posterior view of the thumb.

MRI of the thumb was performed at 3 T (Prisma, Verio, Skyra^fit^, or Skyra, Siemens Healthcare) with a dedicated 16-channel wrist coil (turbo spin echo sequences, FOV, 70–133; matrix, 256–384 × 269–488). The MRI protocol included coronal intermediate-weighted (IW) fat-saturated (FS) (TR/TE, 2580–4800/42–51 ms; slice thickness [ST], 2 mm; echo train length [ETL], 9), sagittal IW FS (TR/TE, 2400–3750/37–67 ms; ST, 2 mm; ETL, 8), axial T2-weighted (TR/TE, 3800–5630/63–83 ms; ST, 2 mm; ETL, 14), and axial T1-weighted (TR/TE, 473–1000/13–14 ms; ST, 2 mm; ETL, 2) images. Additionally, one patient had a computed tomography of the thumb.

### Imaging analysis

All radiographs and MRI studies were retrospectively reviewed by one fellowship-trained musculoskeletal radiologist with more than 7 years of experience (A. H.; Reader 1 [R1]) and one musculoskeletal fellow radiologist (S. M.; Reader 2 [R2]). The two modalities were interpreted independently and randomly with a time interval of 4 weeks.

Prior to the study read-out, four radiographs and MRI were analyzed by both radiologists in consensus to familiarize with the appropriate criteria. These training exams were not part of the present study.

Radiographs were evaluated for the presence or absence of avulsion fractures about the first MCP joint at the following locations: UCL, radial collateral ligament (RCL), checkrein ligaments at the neck of the first metacarpal bone, and phalangoglenoid ligaments/volar plate at the base of the proximal phalanx (Fig. 2).

The distance of dislocated fragments of collateral ligament avulsions from the donor site was measured (mm) on the anteroposterior view in PACS.

MR images were assessed for the presence or absence of a sprain, a partial or complete tear, or an osseous avulsion of the UCL and RCL of the first MCP joint.

A sprain was defined as slight hyperintense signal in IW FS images of and surrounding the ligament without interruption of fibers, a partial tear was defined as an incomplete continuity of the ligament, and a complete tear was defined as interruption of all fibers.

Dislocation was measured from the distal stump to the donor site (mm) for the UCL and RCL.

The presence or absence of a Stener lesion of the collateral ligaments was recorded. A Stener lesion was defined as a displaced ligament proximal and superficial to the adductor aponeurosis.

The accessory UCL and RCL and the dorsal ligamentous complex (capsule, extensor hood, and extensor tendon) were assessed for the presence or absence of a sprain or tear and similar criteria were applied as above. A tear of the transverse intersesamoid fibers was assessed on axial images and confirmed on sagittal images.

Similar to the radiographs, the checkrein ligaments at the neck of the first metacarpal bone and the phalangoglenoid ligaments/volar plate at the base of the proximal phalanx were assessed for the presence or absence of a sprain or tear.

Bone marrow edema pattern about the articulating structures of the first MCP joint was also documented.

### Clinical and intraoperative report review

Data from the electronic medical record were retrieved for trauma history, date of trauma, final clinical diagnosis, and final treatment by the radiology fellow (S.M.).

### Statistical analysis

Descriptive statistics were used to report the imaging interpretation.

A McNemar test was performed to determine statistical difference in frequencies of volar lesions between patients with a dislocated UCL tear and non-displaced UCL tear. A *p* value of < 0.05 was considered statistically significant.

Interrater agreement was assessed using kappa (*κ*) statistics for qualitative data and the intraclass correlation coefficient (ICC) for quantitative data. According to Landis and Koch, a *κ* value of 0–0.20 indicates slight agreement; 0.21–0.40 fair agreement; 0.41–0.60 moderate agreement; 0.61–0.80 substantial agreement; and 0.81–1 almost perfect agreement [17]. The quality of interrater reliability by means of ICC was classified as follows: > 0.75 excellent, 0.4–0.75 fair to good, and < 0.4 poor [9]. For all analyses, statistical software (SPSS version 22, IBM) was used.

## Results

Twenty-five patients (19 men; six women) with a mean age of 37.9 years (SD ± 15.8, range 18–70 years) were included.

Indications for radiographs were sports-related injuries to the thumb (cycling, snowboarding, or skiing). MRIs were performed in patients with clinical suspicion for a dislocated tear of the UCL including Stener lesion and/or first MCP joint instability.

### Imaging analysis

Tables 1 and 2 summarize the imaging findings.

**Table 1:** Injuries at the first metacarpophalangeal joint on radiographs and MRI

Modality	UCL	RCL	Checkrein ligaments	Phalangoglenoid ligaments/volar plate	Volar ligaments	Accessory UCL	Accessory RCL	Extensor hood
Radiographs								
Reader 1	12 (3)	0	36 (9)	12 (3)	44 (11)	n.a.	n.a.	n.a.
Reader 2	12 (3)	0	36 (9)	8 (2)	40 (10)	n.a.	n.a.	n.a.
MRI								
Reader 1	100 (25)	52 (13)	88 (22)	80 (20)	92 (23)	52 (13)	8 (2)	0
Reader 2	100 (25)	32 (8)	88 (22)	76 (19)	96 (24)	48 (12)	8 (2)	0

Data are percentages with absolute numbers in parentheses of 25 patients

An injury is defined as osseous avulsion on radiographs and as sprain, partial, complete rupture, or osseous avulsion on MRI. Accessory UCL and RCL and extensor hood were not assessed on radiographs

*n.a*. not assessed

**Table 2:** Injuries of the ulnar collateral ligament at the first metacarpophalangeal joint on MRI

Reader	Sprain/partial rupture	Complete rupture	Avulsion	Dislocation (≥ 3 mm)	Stener lesion
1	24 (6)	48 (12)	28 (7)	40 (10)	24 (6)
2	24 (6)	60 (15)	16 (4)	56 (14)	20 (5)

Data are percentages with absolute numbers in parentheses of 25 patients

Dislocation of the collateral ligament of ≥ 3 mm includes Stener lesions

### Radiographs

**Fig. 3:**
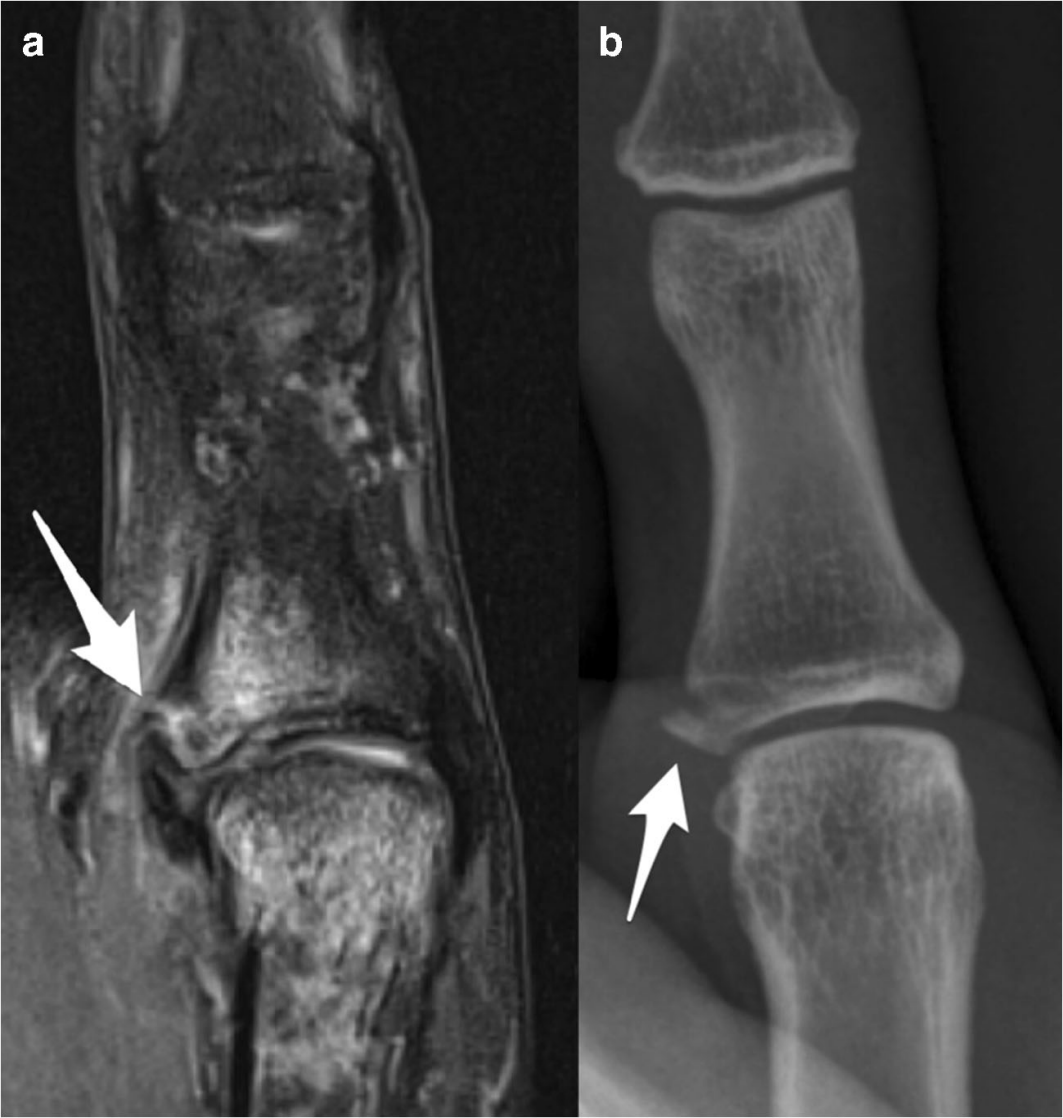
Coronal intermediate-weighted fat-saturated image (**a**) demonstrates a bony avulsion of the distal insertion of the UCL (arrow in **a**). The corresponding palmodorsal radiograph (**b**) shows the minimally displaced fracture at the ulnar base of the proximal phalanx

**Fig. 4:**
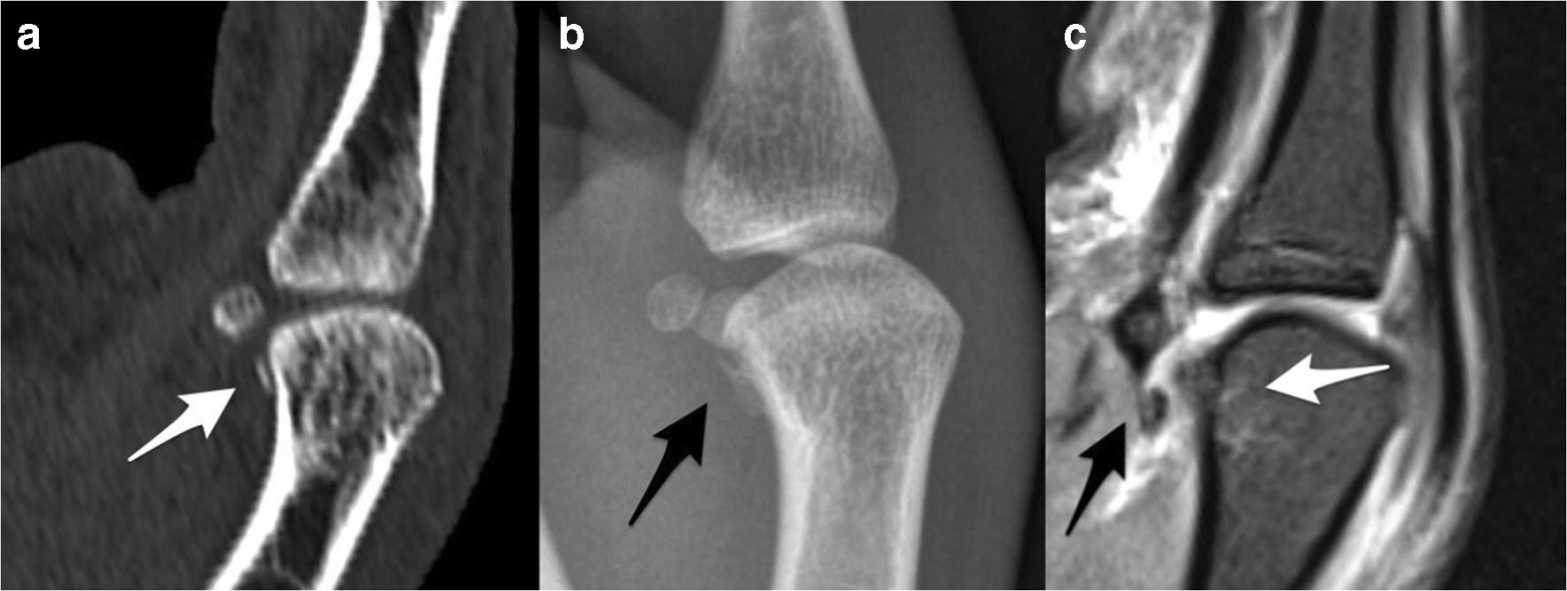
Sagittal reformatted computed tomographic image (**a**), lateral radiograph (**b**), and sagittal intermediate-weighted fat-saturated image (**c**) demonstrate a bony avulsion at the proximal insertion of the checkrein ligament (white arrow in **a**, black arrows in **b** and **c**). Note the subtle bone marrow edema pattern (white arrow in **c**) at the volar aspect of the metacarpal head adjacent to the bony avulsion

The majority of radiographs were unremarkable. Avulsion fractures of the UCL were present in only a minority of patients (12% for both readers; Fig. 3). Associated avulsion fractures at the checkrein ligament insertion (36% for both readers) were seen most frequently (Fig. 4). Avulsion at the phalangoglenoid ligament/volar plate insertion was only evident in a minority of patients (12% R1; 8% R2) on radiographs. No avulsion fracture of the RCL was seen.

### MRI

A complete UCL rupture was present in nearly half of the patients (48% R1; 60% R2).

Displaced UCL tears were seen in almost all complete ruptures (83% R1; 93% R2).

**Fig. 5:**
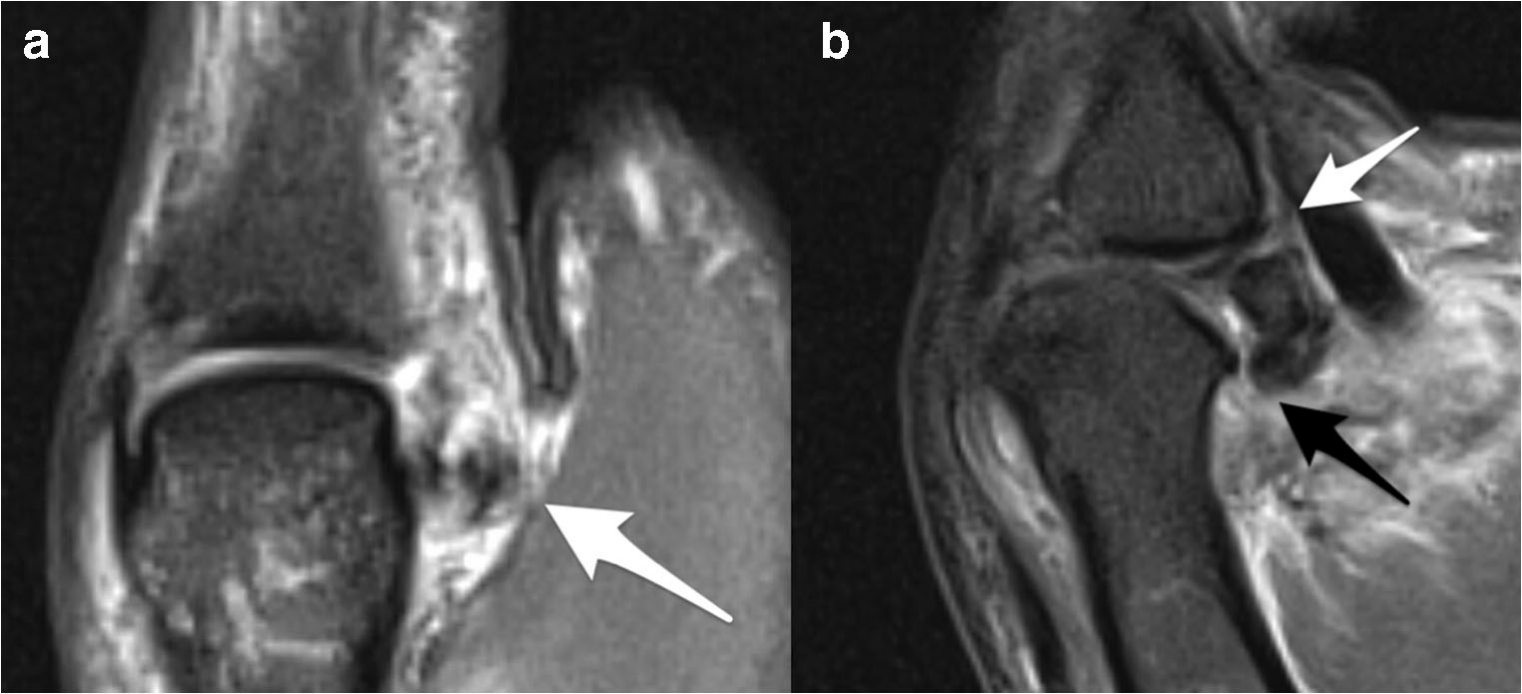
Coronal intermediate-weighted fat-saturated image (**a**) demonstrates a Stener lesion (white arrow). The dislocated distal stump is retracted proximally and wrapped around the adductor pollicis aponeurosis (yo-yo on a string appearance). Sagittal intermediate-weighted fat-saturated image (**b**) demonstrates a concomitant complete disruption of the volar ligaments, the checkrein (black arrow), and phalangoglenoidal ligaments (white arrow)

A Stener lesion was present in 31% (R1) and 26% (R2) of all complete UCL ruptures (Fig. 5).

Twenty-two of the 25 patients (88%) showed an injury to the checkrein ligaments (Fig. 4). Associated injuries to the phalangoglenoid ligaments/volar plate were commonly present on MRI (80% R1; 76% R2).

**Fig. 6:**
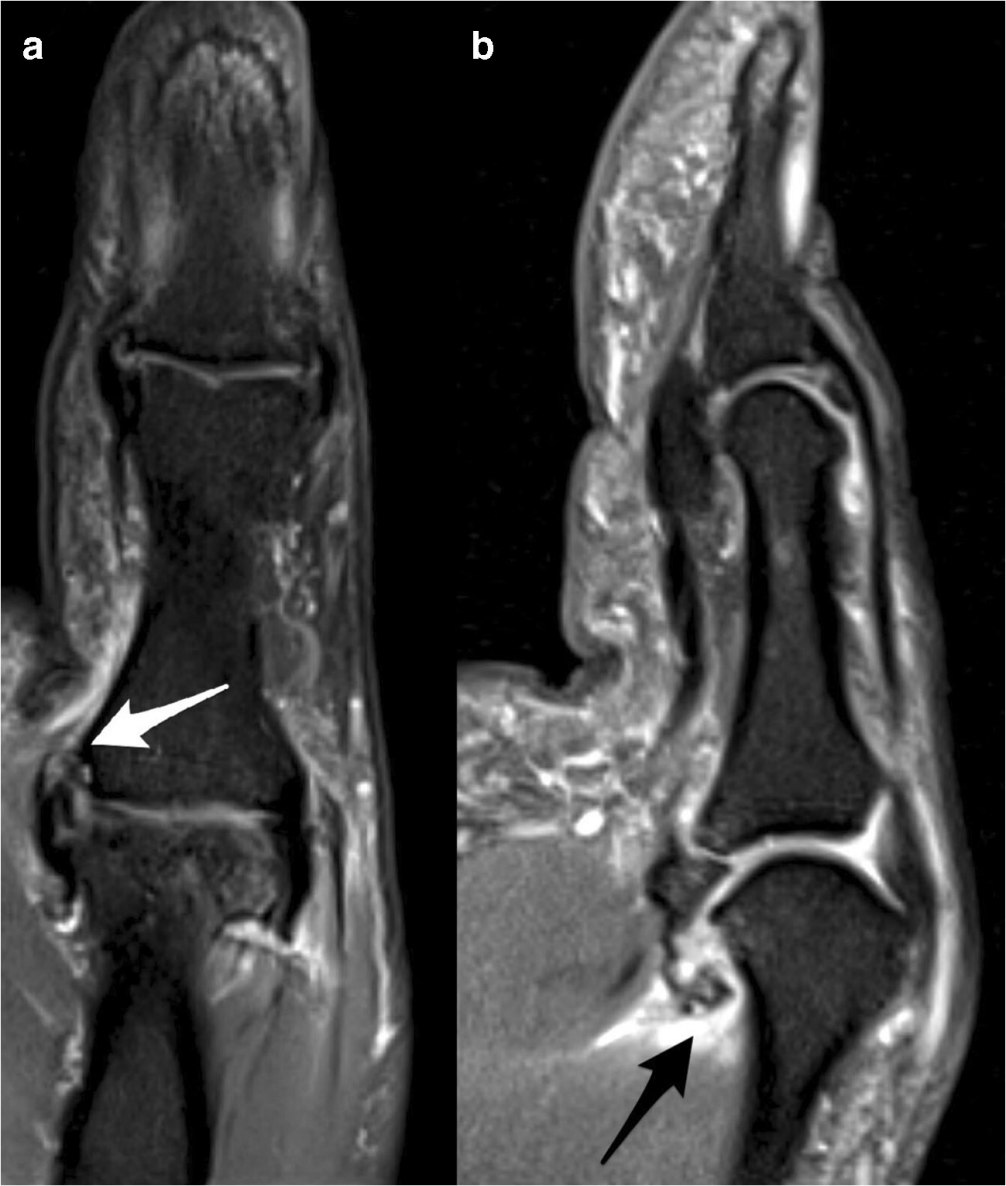
Coronal (**a**) and sagittal (**b**) intermediate-weighted fat-saturated images demonstrate a displaced UCL tear (**a**, white arrow) at the distal insertion of the first MCP joint and a concomitant volar ligament injury at the proximal insertion of the checkrein ligaments (**b**, black arrow)

All patients with a dislocated UCL tear showed a concomitant volar ligament injury (phalangoglenoid/checkrein ligaments or volar plate) for both readers, and even two-thirds of the non-displaced UCL tears (66% [10/15] R1; 73% [8/11] R2) had an injury to the volar ligaments (Fig. 6). No significant difference was found between dislocated and non-dislocated UCL tears in patients with volar ligament injuries (*p* = 0.648).

Complete tears of the RCL were not frequently present (16% [4/25] R1; 12% [3/25] R2). However, the majority of RCL tears were dislocated of more than 3 mm (75% [3/4] R1; 67% [2/3] R2). No injury to the dorsal ligamentous complex was seen.

Bone marrow edema pattern at the distal first metacarpal bone (76% [19/25] R1; 60% [15/25] R2) and at the base of the proximal phalanx (60% [15/25] R1; 56% [14/25] R2) was commonly present.

### Clinical and intraoperative report review

Seven patients underwent surgery due to clinically instability. Surgical repair of the UCL was recommended by the surgeons in another five patients, of whom three declined the procedure and two were lost for follow-up.

Patients with an intraoperative proven Stener lesion (5/25) were surgically repaired; sprains and partial ruptures of the UCL (6/25) according to the clinical chart were treated with immobilization in a cast for 4 to 6 weeks.

Treatment of complete UCL ruptures or avulsions (14/25) varied and was determined individually for each patient depending on joint stability, pain, imaging results, and patient’s requests. Two of them were surgically repaired. The surgeons performed a refixation of the UCL in six (24%) patients and in one (4%) patient an additional surgical repair of the volar ligaments due to failed conservative treatment with persistent joint instability after immobilization for 4 weeks.

The majority (7/9 for both readers) of patients with a clinical indication for a surgical repair had a dislocated UCL of more than 3 mm on MRI.

The surgical findings of five Stener lesions and two complete ruptures of the UCL without a Stener lesion were correctly reviewed by both readers.

### Interrater agreement

On radiographs, interrater agreement was perfect for UCL avulsion fractures (*κ* = 1.0), almost perfect for avulsion fractures of the checkrein ligaments (*κ* = 0.83), and substantial for avulsion fractures of the phalangoglenoid ligaments/volar plate (*κ* = 0.78).

On MRI, interrater agreement was substantial for UCL and phalangoglenoid ligaments/volar plate injuries (*κ*, 0.67 and 0.65, respectively) as well as for Stener lesions (*κ* = 0.78) and moderate for injuries at the RCL (*κ* = 0.60), accessory UCL (*κ* = 0.60), and accessory RCL (*κ* = 0.457).

The evaluation of injuries of the checkrein ligaments at MRI showed poor agreement (*κ* = − 0.136).

The ICCs for measurements of the UCL dislocation on MRI (0.919) and radiographs (0.938) were excellent.

## Discussion

Following an acute trauma of the first MCP joint, we demonstrated that the presence of an injury to the UCL, assessed on MRI, was associated with an additional rupture of the checkrein ligaments in 88% of patients and the phalangoglenoid ligaments/volar plate in about 80%. Associated injuries to the volar structures of the thumb have been repeatedly reported; however, the prevalence of these associated injuries has never been investigated [5, 10, 14].

Post-traumatic hyperextension with an unrecognized or improperly treated tear of the volar ligaments may lead to instability. However, a laxity of the thumb in sagittal plane causes rarely an impairment of the patient and is more often seen in the context of a generalized ligamentous laxity [6]. It seems that a surgical treatment is only recommended for cases with significant instability and failing conservative therapy. Maybe this is an explanation that only one patient in our cohort underwent a refixation of the volar plate.

Palmer and Louis showed in 20 thumb cadavers that the first MCP joint is grossly unstable in any position by radial stress after cutting the UCL and volar ligaments [7]. The thumb is most important for grasping and pinching. Patients with a thumb instability possess weakness in grasping as they are not able to volitionally maintain the joint in flexion [11]. The failure to diagnose and treat these injuries can result in pain, stiffness, and deformity and may lead to early osteoarthritis [12]. There are several surgical procedures such as a volar capsulodesis or extensor pollicis brevis autograft to correct hyperextension of the MCP joint following traumatic injuries with good outcome, even of up to 5 years following injury [6, 13]. Volar injuries to the digits 2–5 have been reported to be commonly seen on radiographs [12]. However, at the first metacarpophalangeal joint, volar avulsion fractures may often be surmised, as was found in our study, most likely due to the convex shape of the metacarpal head and the ulnar and radial sesamoid bones, which both may obscure subtle avulsion fractures. Radiographs are inexpensive and an easy available first-line imaging modality to evaluate potential avulsion injuries or joint incongruences; however, often radiographs do not show associated findings that are important for appropriate treatment decision.

MRI is the imaging method capable to assess all different structures around the joint including the ligaments, tendons, and bone with detailed assessment of injury severity [18, 19]. Sensitivity and specificity in detecting UCL tears of the first MCP joint have been reported to be 100% using MRI [19] with excellent interrater agreement [20], similar to our substantial agreement on UCL tears and associated volar ligament injuries. As an exception, in our study, the agreement for the checkrein ligaments was poor most likely due to the high amount of ruptured ligaments and only three intact ligaments, which were mismatched by the two readers.

However, MRI is not routinely performed as it is costly and not promptly available in most institutions. Most likely due to this reason, data on the prevalence of associated injuries are sparse.

Ultrasound may play an important role with the benefit of ligament stress testing during examination with clear visualization of a ligament discontinuity, especially in chronic injuries.

However, major disadvantage is the observer dependency. Several studies showed that ultrasound is highly dependent on the musculoskeletal experience of the investigator and, with this, less accurate and standardized than MRI [15, 16]. The thin volar ligaments may be challenging to visualize using ultrasound; however, volar joint instability may be an indirect finding of an injury to the volar ligaments. However, until today, no validated data have been published investigating the benefit of ultrasound in volar ligamentous injuries of the first MCP joint.

We also found that a dislocation of the UCL of more than 3 mm is associated with clinically unstable first MCP joint, underlining the need for a surgical repair [1]. The majority of patients in our study undergoing ligament refixation had a dislocation of the UCL of more than 3 mm. This indicates that a significant dislocation of the UCL accompanies joint instability and the length of dislocation should be provided in the radiology report as advocated by Milner et al. [1]. Contrary to the literature, we did not find any avulsion fracture or tear to the dorsal ligamentous structures of the first MCP joint in our cohort [14].

Some limitations should be acknowledged including the retrospective nature of our study, a rather small study group, and lack of surgical confirmation of the diagnosis in all patients. A selection bias may be present in our study, as we only included patients with MRI and radiographs following thumb trauma, indicating a more severe injury.

In conclusion, following traumatic UCL lesions, MRI detects a high percentage of volar ligament injuries that are often surmised on radiographs. Severely dislocated UCL tears of ≥ 3 mm, including Stener lesions, are more often associated with volar ligament injuries.

## Abbreviations

FS: Fat-saturated
ICC: Intraclass correlation coefficient
IW: Intermediated-weighted
MCP joint: Metacarpophalangeal joint
MRI: Magnetic resonance imaging
RCL: Radial collateral ligament
ST: Slice thickness
UCL: Ulnar collateral ligament
